# Hippocampal subfield and amygdala volumes are associated with difficulties in emotion regulation of depressed patients with a history of childhood maltreatment

**DOI:** 10.3389/fpsyt.2025.1641745

**Published:** 2025-08-25

**Authors:** Mónika Gálber, Szilvia Anett Nagy, Gergely Orsi, Gábor Perlaki, Tamás Tényi, Boldizsár Czéh, Maria Simon

**Affiliations:** ^1^ Neurobiology of Stress Research Group, Szentágothai Research Centre, University of Pécs, Pécs, Hungary; ^2^ HUN-REN-PTE Clinical Neuroscience MR Research Group, Pécs, Hungary; ^3^ Department of Neurology, Medical School, University of Pécs, Pécs, Hungary; ^4^ Pécs Diagnostic Centre, Pécs, Hungary; ^5^ Department of Neurosurgery, Medical School, University of Pécs, Pécs, Hungary; ^6^ Department of Psychiatry and Psychotherapy, Medical School, University of Pécs, Pécs, Hungary; ^7^ Department of Laboratory Medicine, Medical School, University of Pécs, Pécs, Hungary

**Keywords:** adverse childhood experiences, child abuse, emotional processing, hippocampus, magnetic resonance imaging, MRI, major depressive disorder, volumetry

## Abstract

**Background:**

Previous studies indicate that hippocampal (subfield) and amygdala volumes may correlate with specific cognitive functions, coping strategies and emotion regulation. Here, we investigated associations between emotional processing and volumes of hippocampal subfields and amygdala. We focused on depressed patients since emotional dysregulation and hippocampal volume shrinkage are characteristic of them. Our hypothesis was that in depressed individuals, maladaptive emotional behaviors will correlate with hippocampal and amygdala volume shrinkage.

**Methods:**

We recruited depressed patients with a history of childhood maltreatment (n=21), depressed patients without maltreatment (n=18), and matched controls (n=21). Their brains were imaged with magnetic resonance imaging and area reconstruction was performed with the FreeSurfer software. History of maltreatment was assessed with Childhood Trauma Questionnaire (CTQ). Emotion processing difficulties were evaluated using the Cognitive Emotion Regulation Questionnaire (CERQ), Difficulties in Emotion Regulation Scale (DERS), Toronto Alexithymia Scale (TAS) and Reading the Mind in the Eyes Test (RMET).

**Results:**

Depressed patients, especially maltreated subjects had small, but nonsignificant hippocampal and amygdala volume decrease (≤10%) and displayed pronounced difficulties in emotion regulation. In maltreated individuals, we found positive correlations between CERQ–rumination and volume of the right CA3, as well as between CERQ–positive-reappraisal and volume of the left presubiculum. In maltreated individuals, CTQ–emotional-abuse scores showed positive correlation with amygdala volumes of both hemispheres. In non-maltreated depressed patients, we found negative correlations between CERQ–rumination and volumes of the right hippocampus and amygdala, as well as several subfields of the right hippocampus. Furthermore, in non-maltreated depressed patients, CTQ–emotional-neglect had a positive correlation with the volume of the right CA3. Overall, among the tests, CERQ–rumination scores had the largest number of correlations with hippocampal subfield volumes mainly in non-maltreated depressed subjects. We found no correlation between alexithymia and brain area. Amygdala volumes had very few correlations, and only with CERQ and CTQ scores.

**Limitations:**

Relatively small sample size, cross-sectional design, retrospective self-report questionnaire to assess adverse childhood experiences and no amygdala subnuclei segmentation.

**Conclusions:**

We could not confirm our hypothesis that maladaptive emotional behavior is associated with hippocampal volume shrinkage. Future studies should preferably focus on functional neuroimaging when examining complex emotional phenomena.

## 1 Introduction

The hippocampal complex plays a vital role in the formation and retrieval of declarative episodic memories, as well as in spatial learning and navigation. Besides these well-documented functions, the hippocampus has a significant role in social cognition and behavior (1, 2). In the context of emotional situations, it interacts with the amygdala (3), and together, they are key integrators of emotion and cognition, a function that is particularly vulnerable in mental disorders (4, 5). Furthermore, functional magnetic resonance imaging (MRI) studies provide direct evidence that the hippocampus is a crucial component of the emotional brain network and play a vital role in emotion processing (6).

Neuroanatomists divide the human hippocampal formation into several subfields, such as the dentate gyrus (DG), Cornu Ammonis (CA1, CA2, CA3, and CA4), and the subicular complex. Additionally, several further dimensions exist, e.g. the medial-lateral and longitudinal dimensions (7). Specific functions are attributed to each subfield, for example, numerous roles in learning and memory are accredited to the DG (8, 9), the CA3 area is important for the rapid encoding of memory (10) and in encoding of new spatial information within the short-term memory (11). Furthermore, a special role in social recognition memory is accredited to the CA2 region (12).

There has been ongoing interest in measuring the volumes of different hippocampal subfields and correlating them with disease pathology or with various aspects of cognitive and emotional regulation (e.g. 13–19). We should however emphasize that correlating brain area volumes with psychological functioning is a controversial scientific approach since experiments that aim to link morphology with complex behavior often yield ambiguous results (15). There is a widely held notion in the literature that hippocampal volume can be linked to cognition and that reduced hippocampal volume due to aging or a mental disorder such as schizophrenia, depression or post-traumatic stress disorder, results in hippocampal dependent cognitive deficits. However, there is clinical evidence that confront this notion of causality and raise the possibility that pre-determined inter-individual differences in hippocampal volume may in fact determine the vulnerability to psychopathology or age-related cognitive impairments (20, 21).

The aim of our current study was to further investigate potential correlations between the volumes of hippocampal subfields and the amygdala in relation to emotional processing. To examine these questions, we focused on patients with major depressive disorder (MDD) who have a history of childhood maltreatment (CM). We studied this population since they typically have difficulties with emotion regulation, and changes in hippocampal and amygdala volumes are often observed in these individuals (17, 22–30).

Participants of the present study completed five psychological questionnaires. Four of these assessments have been specifically developed to measure difficulties in regulating or recognizing emotions. The Difficulties in Emotion Regulation Scale (DERS) assessed the severity of emotional dysregulation. The Cognitive Emotion Regulation Questionnaire (CERQ) was used to evaluate the cognitive coping strategies employed in response to stressful life events. The presence and severity of alexithymia were assessed with the 20-item Toronto Alexithymia Scale (TAS). To assess the ability to identify facial emotional expressions, we utilized the Reading the Mind in the Eyes Test (RMET), which displays only the eye region of the face expressing complex emotions. Finally, the Childhood Trauma Questionnaire (CTQ) was employed to assess participants’ history and severity of childhood abuse and neglect.

Emotion dysregulation is a fundamental feature of mood disorders. In this study, we utilized the DERS, a widely recognized self-report questionnaire designed to assess individuals’ difficulties in recognizing and managing negative emotions (31). This scale has been proven to be reliable in research involving psychiatric patients (32). Individuals, who have experienced childhood maltreatment, often face difficulties with emotion regulation (33). Furthermore, challenges in emotion regulation have been linked to the volumes of hippocampal subfields (19, 34).

The CERQ is a widely used multidimensional tool constructed to identify the cognitive coping strategies in response to negative life events (35). Recent studies suggest that the volumes of hippocampal subfields are positively associated with the use of some specific coping strategies for cognitive emotional regulation in healthy individuals and in patients with mild cognitive impairment (19, 36).

The TAS had been specifically developed to assess alexithymia (37, 38). Although the test is not without controversy, existing evidence suggests that it is a reliable and valid instrument for measuring deficits in emotional awareness and expression (39). Several neuroimaging studies have explored the relationship between alexithymia and gray matter volume of brain areas involved in emotion processing; however, the neuroanatomical basis of alexithymia remains unclear, as previous studies yielded contradictory findings (40). For example, an early study involving healthy volunteers found that individuals with high levels of alexithymia had less gray matter volume in the amygdala and several other emotion-relevant brain areas (41). In contrast, another study indicated a positive association between alexithymia and amygdala volume (42). More recently, a study comparing depressed patients with control subjects reported that higher alexithymia scores were linked to decreased grey matter volume of the fusiform gyrus in depressed individuals, while the opposite was found in healthy controls (43). Overall, a meta-analysis of the available data concluded that individuals with high levels of alexithymia consistently exhibited smaller volumes of the left insula, left amygdala, orbital frontal cortex and striatum (40).

The Reading the Mind in the Eyes Test is a widely recognized assessment tool to evaluate theory of mind (ToM) abilities, i.e. the capacity to represent other people’s mind (“mentalizing”) (44). However, this concept has been challenged by researchers who argue that this test relies heavily on the recognition of facial emotional expression which is often impaired in individuals with alexithymia, and several studies indicate a correlation between greater levels of alexithymia and poorer performance on the RMET (45, 46). Notably, impaired performance on the RMET has been documented in abused children (47), as well as in adults with a history of childhood adversity (48), and in depressed patients with adverse childhood experiences (49–51). Furthermore, several neuroimaging studies explored the relationship between brain structure and RMET performance (52–55). Some of these studies found that larger volumes of the amygdala and/or hippocampus were associated with better performance in the RMET (52, 54).

The Childhood Trauma Questionnaire, developed by Bernstein and co-workers in 2003, is one of the most widely used and validated tools for assessing childhood maltreatment. This retrospective self-report questionnaire evaluates various types of childhood maltreatment, categorized into five dimensions: emotional, physical, sexual abuse, and emotional and physical neglect (56). Although there is a debate about the best methods to measure childhood maltreatment, a recent critical appraisal of the available 52 instruments concluded that CTQ is the only scale that has been thoroughly investigated and demonstrated a strong level of evidence with adequate internal consistency, reliability, content validity, structural validity, and convergent validity (57).

The aim of the present study was to further explore the putative associations between emotion processing and volumes of hippocampal subfields and amygdala. To address these questions, we recruited MDD patients with or without a history of childhood maltreatment and compared their data to that of healthy individuals, who had never experienced a depressive episode. We formulated three hypotheses: 1) patients with MDD, especially the maltreated individuals, will have reduced hippocampal volumes; 2) they will exhibit maladaptive emotion processing, and 3) this maladaptive emotion processing will correlate with the volume reduction of the hippocampus and/or the amygdala.

## 2 Materials and methods

### 2.1 Study design and participants

This cross-sectional study involved a total of 60 subjects (40 females). The age range of the subjects was between 18 and 54 years (mean ± SD = 33.5 ± 8.512). Participants diagnosed with MDD were recruited from the Affective Disorder Unit of the Department of Psychiatry and Psychotherapy, Clinical Centre, University of Pécs. Participants with MDD were categorized into two subgroups based on their history of childhood maltreatment (CM). The MDD+CM group included those with moderate to severe CM (N = 21; 14 females), while the non-maltreated MDD group consisted of individuals with a low incidence of childhood maltreatment (N = 18, 12 females). Additionally, a healthy control (HC) group was formed, consisting of subjects matched in age and IQ, with no history of mental disorders (N = 21, 14 females). In the HC group, the Symptom-Checklist-90-R (58, 59) was applied to rule out subthreshold psychiatric symptoms. The detailed demographic data of the three experimental groups are presented in **Table 1**.

**Table 1:** Demographics and assessments of childhood maltreatment and neuropsychiatric status.

Characteristics	MDD+CM (N = 21)	MDD (N = 18)	HC (N = 21)	Between-group differences
Age [mean ± SD (range) in years]	32.90 ± 9.29 (18-54)	34.06 ± 7.60 (21-49)	33.62 ± 8.39 (21-48)	F = 0.052; p = 0.950 ^†^
Number of females (%)	14 (66%)	12 (66%)	14 (66%)	χ^2^ = 0.071 p = 0.965 ^¢^
Years of education (range)	12 (11-15) *	12 (12-17)	15 (12-17)	χ^2^ = 7.222; p = 0.026 ^§^; *post hoc* MDD+CM *vs* HC p = 0.036; MDD *vs* HC p = 1.000; MDD+CM *vs* MDD p = 0.111
IQ (mean ± SD)	112.1 ± 5.6	114.7 ± 4.8	111.9 ± 5.7	F = 0.223, p = 0.810 ^†^
CTQ sum	58 (52-72) *** ^###^	33 (28.8-37)	28 (26.5-33)	χ^2^ = 41.795; p < 0.001 ^§^; *post hoc* MDD+CM *vs* HC p < 0.001; MDD *vs* HC p = 1.000; MDD+CM *vs* MDD p < 0.001
CTQ physical neglect	10 (8-13) *** ^###^	5 (5-7)	5 (5-5)	χ^2^ = 38.636; p < 0.001 ^§^; *post hoc* MDD+CM *vs* HC p < 0.001; MDD *vs* HC p = 0.550; MDD+CM *vs* MDD p <0.001
CTQ physical abuse	9 (6.5-12) *** ^###^	5 (5-5)	5 (5-5)	χ^2^ = 28.952; p < 0.001 ^§^; *post hoc* MDD+CM *vs* HC p < 0.001; MDD *vs* HC p = 1.000; MDD+CM *vs* MDD p < 0.001
CTQ emotional neglect	18 (16-20) *** ^###^	10 (7-12)	8 (6-10.5)	F = 72.997; p < 0.001 ^†^; *post hoc* MDD+CM *vs* HC p < 0.001; MDD *vs* HC p = 0.323; MDD+CM *vs* MDD p < 0.001
CTQ emotional abuse	18 (11.5-20) *** ^###^	7 (5-8)	6 (5.5-8)	χ^2^ = 36.167; p < 0.001 ^§^; *post hoc* MDD+CM *vs* HC < 0.001; MDD *vs* HC p = 1.000; MDD+CM *vs* MDD p < 0.001
CTQ sexual abuse ^a^	5 (5-9.5) *	5 (5-5)	5 (5-5)	U = 128.000; p = 0.014 ^&^
Beck Depression Inventory	23 (17.5-28.5) ***	22 (18-24) ***	4 (2-6)	χ^2^ = 41.045; p < 0.001 ^§^; *post hoc* MDD+CM *vs* HC p < 0.001; MDD *vs* HC p < 0.001; MDD+CM *vs* MDD p = 0.592
Beck Anxiety Inventory	21 (16-33) ***	18 (8-24) ***	3 (0.5-10.5)	χ^2^ = 30.916; p < 0.001 ^§^; *post hoc* MDD+CM *vs* HC p < 0.001; MDD *vs* HC p < 0.001; MDD+CM *vs* MDD p = 0.151
Age at illness onset	21 (16.5-33)	29 (18-34)	-	U = 242.500; p = 0.243 ^&^
Length of illness (years)	7 (0.3-13)	5 (1-7)	-	U = 180.000; p = 0.597 ^&^
Number of MDD episodes	2 (1-3)	2 (1-2)	-	U = 147.000; p = 0.129 ^&^

Data are expressed as median (interquartile range) except age, number of females and IQ.

Benjamini-Hochberg correction was not applied here, because this approach can increase the type II error and may result in elimination of the group-specific differences.

MDD+CM, major depressive disorder with childhood maltreatment; MDD, major depressive disorder; HC, healthy control; IQ, intelligence quotient; CTQ, Childhood Trauma Questionnaire; *vs*, versus.

^†^One-way ANOVA with Bonferroni *post hoc* test; ^¢^Chi-square test; ^§^Kruskal-Wallis H test with Dunn’s pairwise *post hoc*; ^&^Mann-Whitney U test; ^a^ Comparison was only made between the MDD+CM and MDD groups, as no one in the control group gave a positive response that could indicate sexual abuse. *p < 0.05, ***p < 0.001 *versus* healthy controls; ^###^p < 0.001 *versus* the MDD group.

The exclusion criteria for participation were as follows: current substance use (abstinence for < 2 years); IQ < 85; a history of head injury; a history of any neurological or psychiatric disorders (non-excluding psychiatric disorders are summarized below); experience of traumatic life events meeting DSM-5 post-traumatic stress disorder (PTSD) criterion A; or any contraindications for MRI (i.e. claustrophobia or the presence of metal objects in the body).

In our study, non-excluding, co-morbid psychiatric disorders were: anxiety disorders (panic disorder N = 3; generalized anxiety disorder N = 3; social phobia N = 2; specific phobias N = 4); cluster C personality disorders (dependent N = 2, avoidant N = 2); obsessive-compulsive disorder in the past 6 years, and never treated when symptomatic before (N = 1); lifetime sedatives, hypnotics, and anxiolytics use disorder (N = 2) in full remission for more than 2 years; mild and non-chronic alcohol use disorder (N = 2).

In patients with MDD, the mean age of disease onset was 25.49 ± 9.47 years. The mean duration of illness was 7.15 ± 7.74 years (range 0.2–26 years). Thirty-six (97%) patients with MDD were treated with antidepressant medication: SSRIs (N = 25); SNRIs (N = 3); NaSSAs (N= 7); agomelatine (N = 4); trazodone (N= 2); combined with mood stabilizer (N = 2); combined with low-dose atypical antipsychotics (N = 5).

The local Research Ethics Committee of the University of Pécs approved the study design and protocol (Ethical Approval Nr.: 2015/5626). All participants were Hungarian speaking Caucasians, living in the urban and suburban area of Pécs, and gave written informed consent.

### 2.2 Neuropsychiatric assessments, psychological tests, and questionaries

All participants underwent comprehensive screening for any current or past psychiatric disorders, along with an assessment of general intelligence and emotional coping strategies and recognition using standardized neuropsychological measures.

Participants diagnosed with MDD fulfilled the DSM-5 diagnostic criteria for MDD (60), as evaluated by a trained psychiatrist (MS) using the Structured Clinical Interviews for DSM-5 disorders (SCID-5-CV and SCID-5-PD; 61, 62). The 21-item version of the Hamilton Depression Rating Scale (63) and the Beck Depression Inventory (64) were employed to evaluate the severity of depression, while the Beck Anxiety Inventory (65) was used to examine the severity of anxiety. Participants were also evaluated using the 11-item General Traumatic Experiences subscale of the 21-item Self Report Early Trauma Inventory (66) to measure causal traumatic childhood life events. Individuals with random trauma were excluded from this study. Four-subtest version of Hungarian adaptation of the Wechsler Adult Intelligence Scale-Revised was applied to test the General Intelligence Quotient (IQ; 67–69). See data for the results of the psychiatric assessments in **Table 1**.

#### 2.2.1 Assessment of childhood maltreatment: Childhood Trauma Questionnaire

The history and severity of chronic and/or repeated childhood maltreatment were assessed using the self-reported, retrospective, 28-item Childhood Trauma Questionnaire (CTQ; 56). Participants had to fill out the Hungarian version of the CTQ (70, 71).

The CTQ evaluates the severity of five distinct types of maltreatment experienced prior to the age of 18: physical neglect (PN), physical abuse (PA), emotional neglect (EN), emotional abuse (EA), and sexual abuse (SA). Each subscale comprises 5 items, which participants evaluate using a 5-point Likert scale. In the present study, participants were enrolled in the MDD+CM subgroup if they obtained CTQ scores that exceeded the established cut-off values of the “low” range on any of the CTQ subscales. The cut-off values (i.e. maximum score) of the “low” range for the various subscales were as follows: physical neglect: 9; physical abuse: 9; emotional neglect: 14; emotional abuse: 12; sexual abuse: 7. For a detailed description of the assessment of childhood maltreatment see our earlier publications (50, 72, 73). A detailed summary of the CTQ results is presented in **Table 1**.

#### 2.2.2 Assessment of cognitive coping strategies in response to stressful life events: Cognitive Emotion Regulation Questionnaire

CERQ is a widely used test for assessing specific emotion regulation strategies in response to threatening or stressful life events (35). We used the Hungarian version of the 36-item CERQ (74). It consists of 9 subscales, each of which represents separable emotion regulation strategies one can deploy: 1) self-blame; 2) rumination; 3) catastrophizing; 4) blaming others; 5) acceptance; 6) positive refocusing; 7) refocus on planning; 8) positive reappraisal; 9) putting into perspective, each subscale consists of 4 items.

Subscales 1, 2, 3, and 4 are categorized as maladaptive strategies because they can hinder an individual’s ability to cope effectively with stressful events. In contrast, subscales 5, 6, 7, 8, and 9 are classified as adaptive cognitive strategies that can enhance effective coping with stressful situations. Participants rated each item on a 5-point Likert scale, where 1 signifies “rarely” and 5 signifies “almost always.” Higher scores on each subscale indicate a greater tendency to use that particular emotion regulation strategy.

We calculated the “CERQ maladaptive sum” by aggregating the scores of Self-Blame, Rumination, Catastrophizing, and Blaming Others subscales. The rigid use of these maladaptive strategies may increase vulnerability to stressful life events. Therefore, they are typically associated with a range of mental health problems, including depression, and can worsen the clinical outcomes (75). Brief interpretations of these subscales are listed here. 1) Self-Blame: Frequently blaming oneself for negative events, which can lead to feelings of guilt, shame, and diminished self-esteem. 2) Rumination: The tendency to constantly dwell on negative thoughts and feelings related to a negative event. This strategy can intensify negative emotions and hinder recovery. 3) Catastrophizing: Magnifying the severity and negative consequences of an adverse event, potentially leading to heightened anxiety and distress. 4) Blaming Others: Frequently blaming others or external factors for negative events. This strategy may undermine personal responsibility and effective problem-solving. Higher scores on these maladaptive subscales suggest a greater reliance on strategies that may be detrimental to mental health.

We also calculated the “CERQ adaptive sum” which was obtained by summing the scores of Acceptance, Positive Refocusing, Refocusing on Planning, Positive Reappraisal, and Putting into Perspective subscales. The flexible implementation of adaptive emotion regulation strategies is typically associated with resilience or with a better clinical outcome. The interpretation of these adaptive strategies are as follows. Acceptance: Coming to terms with the negative event and accepting that it has happened. Positive Refocusing: Shifting attention away from negative thoughts towards more pleasant and positive thoughts. Refocus on Planning: Focusing on developing a plan of action to deal with the negative event. This can promote a sense of control and proactive problem-solving. Positive Reappraisal: Finding a positive meaning in a negative event which can promote personal growth and resilience. Putting into Perspective: Downplaying the significance of a negative event by comparing it to more significant negative events. Higher scores on adaptive subscales suggest a greater tendency to use strategies that promote positive emotional well-being.

#### 2.2.3 Assessment of difficulties in emotion regulation: Difficulties in Emotion Regulation Scale

DERS is a widely used self-report questionnaire designed to measure challenges in emotion regulation among adults (31). Participants completed the Hungarian version of the DERS and when they reached higher scores that indicated greater difficulties (76). The scale consists of 41 items divided into six subscales: 1) Nonacceptance of Emotional Responses (DERS–nonacceptance), 2) Difficulties Engaging in Goal-Directed Behavior (DERS–goals), 3) Impulse Control Difficulties (DERS–impulse), 4) Lack of Emotional Awareness (DERS–awareness), 5) Limited Access to Emotion Regulation Strategies (DERS–strategies), and 6) Lack of Emotional Clarity (DERS–clarity).

Brief interpretations of these subscales are given here. 1) Nonacceptance of Emotional Responses: High scores on this subscale indicate that an individual may struggle with the acceptance of negative emotions, potentially leading to increased distress and difficulties in managing those emotions effectively. 2) Difficulties Engaging in Goal-Directed Behavior: Elevated scores suggest that individuals may experience challenges in maintaining focus and engaging in goal-oriented activities during periods of distress. 3) Impulse Control Difficulties: High scores suggest a tendency to impulsive actions or unconsidered reactions when experiencing emotional distress, which can increase existing challenges. 4) Lack of Emotional Awareness: This subscale measures the extent to which individuals are aware of their own emotional states. High scores indicate a deficiency in self-awareness or an unwillingness to acknowledge their emotions. 5) Limited Access to Emotion Regulation Strategies: High scores indicate a belief that few effective strategies are available to effectively manage negative emotions, leading to a diminished sense of control over emotional reactions. 6) Lack of Emotional Clarity: This subscale evaluates the extent to which individuals understand and can identify their emotions. High scores suggest that individuals may struggle to understand emotional reactions and the underlying reasons for them. Patients rated their experiences to each item on a 5-point Likert scale, where 1 indicates “rarely” and 5 “almost always”.

#### 2.2.4 Assessment of alexithymia: Toronto Alexithymia Scale

The TAS-20 is a test designed to assess alexithymia (37, 38), although it is not without shortcomings (77). The TAS-20 has three subscales: 1) difficulty identifying feelings; 2) difficulty describing feelings; and 3) externally oriented thinking. Items are rated using 5-point Likert scales whereby 1 = strongly disagree and 5 = strongly agree. Participants completed the Hungarian version of the TAS-20 (78).

#### 2.2.5 Assessment of face emotion recognition: Reading the Mind in the Eyes Test

All participants completed the RMET, which is widely used as a measure of social cognitive ability (79). The test comprises 36 photographs of male and female eyes illustrating emotionally charged or neutral mental states. Subjects must select which of four mental-state descriptors best matches what the person in the photograph is thinking or feeling. This test is also regarded as an advanced Theory of Mind test in which participants need to put themselves in the place of the person in the picture (44). We quantified the number of correctly identified facial expressions and classified them into emotionally charged (RMET emotional sum) and neutral expressions (RMET neutral faces). Within the emotionally charged category, we examined separately the correctly recognized negative (RMET negative emotion) and positive emotional expressions (RMET positive emotion).

### 2.3 Volumetric analysis with *in vivo* magnetic resonance imaging

#### 2.3.1 MRI acquisition

Data were collected with a 3T Magnetom Trio TIM MRI scanner (Siemens AG, Erlangen, Germany) using a 12-channel head coil.

For the volumetric measurements, isotropic T1-weighted high-resolution images were obtained using a three-dimensional magnetization prepared rapid acquisition with gradient echo sequence (3D-MPRAGE) with the following parameters: repetition time/inversion time/echo time (TR/TI/TE): 2530/1100/3.37 ms; flip angle: 7°; number of averages: 1; field of view: 256 × 256 mm^2^; matrix size: 256 × 256; 176 sagittal slices with a thickness of 1.00 mm; bandwidth: 200 Hz/pixel.

#### 2.3.2 Image processing

T1-weighted images underwent complex pre- and post-processing before the statistical analysis. First, visual quality control was performed to exclude data containing artifacts. In order to examine the relationship between the results of the psychological tests and the volume of hippocampal subfields and amygdala, cortical and subcortical reconstruction and segmentation were carried out using the FreeSurfer software 6.0 (https://surfer.nmr.mgh.harvard.edu/ 80). Technical details of the automated cortical and subcortical segmentation stream are described in a prior methodological study (81, 82). Talairach transformation and the removal of non-brain tissues were visually verified, and error correction was performed when necessary, based on the recommended workflow (https://surfer.nmr.mgh.harvard.edu/fswiki). Gray matter parcellation was estimated using an automated labelling procedure based on the Desikan-Killiany-Tourville Atlas (83). Segmentation and structure labelling were then confirmed and edited manually using standard procedures recommended by the FreeSurferWiki website, and recon-all was rerun to estimate the volumes.

**Figure 1:**
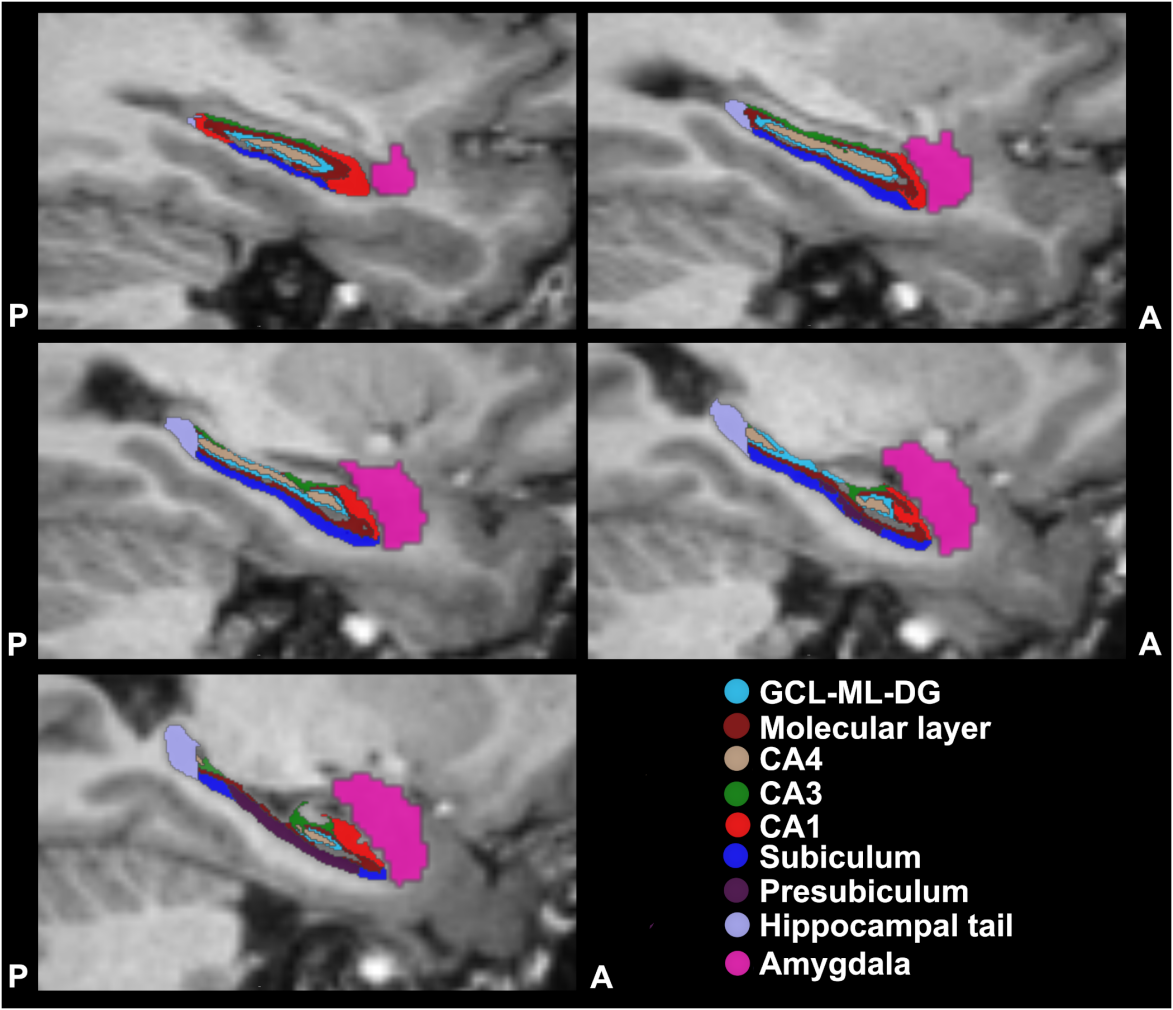
Reconstruction and segmentation of amygdala and hippocampal subfields. Representative T1-weighted images of reconstructed subareas from the left side of the brain. Five slices are shown here in the sagittal plane, in a direction from left to right, i.e. X = -29 to X = - 21 in the Talairach space. The segmentation was based on the ex vivo hippocampal subfield atlas described by Iglesias et al. (84). The different colours indicate different subfield areas. The reconstructed 3D volumes of the different subfields were correlated with the results of the psychological tests. A, anterior; CA, Cornu Ammonis; GCL-ML-DG, granule cell layer and molecular layer of the dentate gyrus; P, posterior.

The following bilateral hippocampal subfields were segmented from T1-weighted images: granule cell layer and molecular layer of the dentate gyrus (GCL-ML-DG), molecular layer, CA4, CA3, CA1, subiculum, presubiculum, parasubiculum, hippocampal-amygdaloid transition area, fimbria, hippocampal tail, and hippocampal fissure (https://surfer.nmr.mgh.harvard.edu/fswiki/HippocampalSubfields; 84). Subfield’s volume having a standard deviation greater than 10% of the mean were excluded from further analysis; therefore, the bilateral parasubiculum, the hippocampal-amygdaloid transition areas, fimbria, and the hippocampal fissures were ruled out. The volume of the entire hippocampus was calculated by the sum of all subregions except the hippocampal fissure, while the volume of the amygdala was obtained from subcortical segmentation output of the FreeSurfer file *aseg.mgz* (see **Figure 1**). Volumes are expressed in mm^3^.

### 2.4 Statistical analysis the demographic and clinical data

IBM SPSS Statistics Software version 29.0.2 was applied for data analyses. The assumptions were tested in all cases. For parametric data, one-way ANOVA with Bonferroni or Dunnett’s T3 (in case of unequal variance) *post hoc* test was used to compare groups for demographic, IQ and clinical variables, as well as for CERQ, DERS, TAS and RMET scores. Nonparametric data and datasets with skewed distributions were compared with the Mann-Whitney U test, as well as with the Kruskal-Wallis H test followed by Dunn’s pairwise *post hoc* comparison.

Given that no sexual dimorphism of hippocampal size is observed after adjusting for head size (85) and considering that the Point-Biserial Correlation revealed a relatively strong multicollinearity between estimated total intracranial volume (eTIV) and gender (r_pb_= -0.519, N = 60, p = 0.00002), age and eTIV were selected as regressors for subsequent statistical analyses.

Volumetric data were analyzed with one-way ANCOVA followed by Bonferroni or Dunnett’s T3 (in case of unequal variance) *post hoc* tests, and with Kruskal-Wallis H test followed by Dunn’s pairwise *post hoc* test to identify differences between groups, while controlling for age and eTIV as covariates.

The interaction effect was examined using a general linear model to determine whether there is a general between-group difference in the association of psychological parameters and brain volumes. Group, age, eTIV, psychological parameters, and the group × psychological parameter interaction term were included as independent variables. Brain volumes were used as dependent variables. If any significant findings arose, further within-group multiple linear regression analyses were conducted, while controlling for age and eTIV, to explore the relationship between volumetric data and psychological parameters. The assumptions of multiple linear regressions were satisfied, as judged by testing for linearity, normality assumptions of the residues, outliers, independence of errors, homoscedasticity, and multicollinearity (86).

The level of significance was set at two-tailed p ≤ 0.05 for all statistical tests. Uncorrected p-values are reported to facilitate comparisons with other studies. However, to address the issue of multiple comparisons, Benjamini-Hochberg correction was applied with q = 0.15 when analyzing volumetric and psychological data. P-values that survived this correction for multiple comparisons are indicated in the tables.

## 3 Results

### 3.1 Demographic data and clinical characteristics

The demographic data and the results of the psychiatric assessments are listed in **Table 1**. Since the individuals were carefully selected for this study, age, gender ratio, and IQ values were comparable in the three experimental groups. Maltreated MDD patients spent significantly shorter time in education compared to controls (*post hoc* test: p = 0.036), but they were similar to the MDD group (**Table 1**).

As a result of the group assignment, the CTQ scores of maltreated MDD patients (MDD+CM group) were significantly higher than those of both the non-maltreated MDD patients (MDD group) and the healthy control (HC) group across all subscales, while CTQ scores of the MDD and HC groups were very similar (**Table 1**). Maltreated and non-maltreated depressed patients showed much higher scores in the Beck Depression and Anxiety Inventories compared to control group. The age at illness onset, the length of illness and the number of depressed episodes were comparable in the two sub-groups of depressed patients.

### 3.2 Hippocampal subfield and amygdala volumes

Representative examples of T1-weighted images and the results of the automatic segmentation of the hippocampal subfields and the amygdala are depicted in **Figure 1**. The results of the volumetric analysis are summarized in **Table 2**. We observed a consistent trend for volume reduction, i.e. a shrinkage of 1-10% in nearly all hippocampal subregions of the depressed patients. However, none of these reductions were statistically significant when compared to the control group. Notably, patients who had been exposed to childhood maltreatment generally displayed smaller volumes compared to both the control group and the non-maltreated MDD group, but again, these differences did not reach statistical significance. The largest observed volume reduction (-10%) occurred in the right presubiculum, right hippocampal tail, and left amygdala of the MDD+CM group (**Table 2**). The least affected hippocampal region was the CA3 area of both hemispheres.

**Table 2:** Hippocampal subfield and amygdala volumes.

Brain areas	MDD+CM	MDD	HC	Between-group differences
Right hippocampus	3320.5 ± 418.7 (-6%; -3%†)	3433.2 ± 337.9 (-2%)	3516.6 ± 294.8	F = 0.881; p = 0.420^*^
Right GCL-ML-DG	283.1 ± 37.7 (-3%; -2%†)	288.9 ± 31.4 (-1%)	292.3 ± 23.9	χ^2^ = 1.036; p = 0.596^§^
Right molecular layer	553.5 ± 74.2 (-4%; -2%†)	570.1 ± 56.6 (-1%)	578.8 ± 50.5	F = 0.271; p = 0.764^*^
Right CA4	240.3 ± 32.1 (-4%; -1%†)	243.7 ± 27.7 (-2%)	249.5 ± 21.6	F = 0.205; p = 0.815^*^
Right CA3	198.6 ± 31.1 (+1%; +1%†)	196.5 ± 33.4 (-0.4%)	197.2 ± 23.2	F = 0.474; p = 0.625^*^
Right CA1	622.5 ± 90.3 (-4%; -4%†)	648.5 ± 65.1 (+0.3%)	646.2 ± 62.9	F = 0.249; p = 0.781^*^
Right subiculum	415.8 ± 53.8 (-6%; -4%†)	433.2 ± 41.0 (-2%)	442.8 ± 52.1	F = 0.825; p = 0.444^*^
Right presubiculum	289.7 ± 39.4 (-10%; -4%†)	301.3 ± 34.2 (-6%)	320.4 ± 41.2	F = 2.699; p = 0.076^*^
Right hippocampal tail	522.1 ± 73.3 (-10%; -4%†)	543.1 ± 69.7 (-6%)	576.5 ± 70.5	F = 2.456; p = 0.095^*^
Left hippocampus	3251.6 ± 386.7 (-6%; -3%†)	3352.8 ± 318.1 (-3%)	3441.6 ± 376.2	F = 0.745; p = 0.480^*^
Left GCL-ML-DG	271.6 ± 35.8 (-7%; -5%†)	285.3 ± 30.7 (-2%)	291.1 ± 33.8	χ^2^ = 3.861; p = 0.145^§^
Left molecular layer	537.5 ± 68.4 (-5%; -2%†)	551.0 ± 53.9 (-2%)	564.7 ± 65.4	F = 0.368; p = 0.694^*^
Left CA4	232.2 ± 29.9 (-7%; -4%†)	241.4 ± 27.4 (-3%)	248.0 ± 30.1	χ^2^ = 3.101; p = 0.212^§^
Left CA3	185.2 ± 25.1 (-1%; -2%†)	188.5 ± 26.9 (+1%)	187.0 ± 27.1	F = 0.210; p = 0.811^*^
Left CA1	596.2 ± 81.9 (-5%; -3%†)	614.0 ± 67.3 (-2%)	625.2 ± 73.2	F = 0.200; p = 0.819^*^
Left subiculum	416.4 ± 59.3 (-5%; +0,1%†)	415.9 ± 38.2 (-5%)	438.4 ± 51.3	F = 1.260; p = 0.292^*^
Left presubiculum	301.8 ± 41.7 (-8%; -4%†)	314.8 ± 39.7 (-4%)	327.8 ± 41.6	F = 1.377; p = 0.261^*^
Left hippocampal tail	513.7 ± 51.5 (-6%; -2%†)	524.1 ± 77.4 (-4%)	544.9 ± 85.1	F = 0.558; p = 0.576^*^
Right amygdala	1589.7 ± 276 (-6%; -4%†)	1661.9 ± 202.0 (-1%)	1683.2 ± 166.0	F = 0.501; p = 0.609^*^
Left amygdala	1536.5 ± 251.8 (-10%; -6%†)	1636.7 ± 145.6 (-4%)	1701.7 ± 228.0	F = 2.314; p = 0.108^*^

Data are expressed as mean ± standard deviation in mm^3^. In parentheses, changes in percentage compared to the control group and compared to depressed patients without childhood maltreatment (†).

MDD+CM, major depressive disorder with childhood maltreatment; MDD, major depressive disorder; HC, healthy control; GCL-ML-DG, granule cell layer and molecular layer of the dentate gyrus; CA, Cornu Ammonis.

^*^One-way ANCOVA; ^§^Kruskal-Wallis H test.

### 3.3 Emotion dysregulation, alexithymia, and emotion recognition

Results of the CERQ, DERS, TAS and RMET tests are presented in **Table 3**. In general, depressed patients, especially the ones with the history of childhood maltreatment, displayed numerous difficulties with emotion processing.

**Table 3:** Results of the CERQ, DERS, TAS and RMET tests.

Psychological parameters	MDD+CM	MDD	HC	Between-group differences
CERQ sum	99 (94-105)	100 (91.75-106)	102 (95.5-106.5)	F= 0.335; p = 0.717 ^†^
CERQ maladaptive sum	49 (43.5-54.5) ***	46 (39.25-49) **	34 (28.5-39.5)	F= 15.208; p < 0.001 ^†^; *post hoc*: MDD+CM *vs* HC, p < 0.001; MDD *vs* HC, p = 0.001; MDD+CM *vs* MDD, p = 0.525
CERQ adaptive sum	50 (45.5-55.5) ***	56.50 (46.5-63.25) **	65 (59.5-73)	F= 10.236; p < 0.001 ^†^; *post hoc*: MDD+CM *vs* HC, p < 0.001; MDD *vs* HC, p = 0.003; MDD+CM *vs* MDD, p = 1.000
CERQ–self-blame	14 (9.5-17.5) **	13.5 (10.75-16.25) **	9 (7.5-11.5)	F= 8.435; p < 0.001 ^&^; *post hoc*: MDD+CM *vs* HC, p = 0.001; MDD *vs* HC, p = 0.004; MDD+CM *vs* MDD, p = 0.955
CERQ–rumination	14 (10.5-17) **	14 (11.75-15.5) *	10 (7.5-13.5)	F= 6.253; p = 0.004 ^†^; *post hoc*: MDD+CM *vs* HC, p = 0.008; MDD *vs* HC, p = 0.015; MDD+CM *vs* MDD, p = 1.000
CERQ–catastrophizing	12 (9.5-14.5) ***	9 (7.75-11.5) *	6 (5-9)	F= 14.369; p < 0.001 ^†^; *post hoc*: MDD+CM *vs* HC, p < 0.001; MDD *vs* HC, p = 0.019; MDD+CM *vs* MDD, p = 0.074
CERQ–blaming others	8 (6-11)	8 (7-10)	8 (5-9)	F= 0.504; p = 0.606 ^†^
CERQ–acceptance	12 (10-14)	11.5 (10-13)	11 (8-12.5)	F= 2.546; p = 0.087 ^†^
CERQ–positive refocusing	8 (8-10)	8 (6-10) *	10 (8-14.5)	F= 4.313; p = 0.018 ^†^; *post hoc*: MDD+CM *vs* HC, p = 0.109; MDD *vs* HC, p = 0.021; MDD+CM *vs* MDD, p = 1.000
CERQ–refocus on planning	12 (9.5-15.5) ***	15 (12.75-16.25)	16 (14.5-20)	F= 8.234; p < 0.001 ^†^; *post hoc*: MDD+CM *vs* HC, p < 0.001; MDD *vs* HC, p = 0.113; MDD+CM *vs* MDD, p = 0.248
CERQ–positive reappraisal	9 (6-13) ***	11 (7-15) ***	16 (14-17.5)	F= 14.110; p < 0.001 ^&^; *post hoc*: MDD+CM *vs* HC, p < 0.001; MDD *vs* HC, p < 0.001; MDD+CM *vs* MDD, p = 0.836
CERQ–putting into perspective	8 (7-9) **	8.5 (7-12) **	13 (9-15)	F= 7.916; p < 0.001 ^†^; *post hoc*: MDD+CM *vs* HC, p = 0.002; MDD *vs* HC, p = 0.006; MDD+CM *vs* MDD, p = 1.000
DERS sum	112 (104-139) ***	94.5 (80.5-112.25) **	62 (54-74)	χ^2^ = 32.183, p < 0.001 ^§^; *post hoc*: MDD+CM *vs* HC, p < 0.001; MDD *vs* HC, p = 0.001; MDD+CM *vs* MDD, p = 0.206
DERS–nonacceptance	19 (13-27.5) ***	15 (11.75-21.25) **	9 (8-11.5)	χ^2^ = 23.839, p < 0.001 ^§^; *post hoc*: MDD+CM *vs* HC, p < 0.001; MDD *vs* HC, p = 0.001 MDD+CM *vs* MDD, p = 1.000
DERS–goals	20 (16.5-22) ***	17.5 (12-21) **	12 (9-14)	F= 14.845; p < 0.001 ^†^; *post hoc*: MDD+CM *vs* HC, p < 0.001; MDD *vs* HC, p = 0.002; MDD+CM *vs* MDD, p = 0.491
DERS–impulse	20 (14-23.5) ***	16.5 (11.5-23) **	9 (7-11)	χ^2^ = 25.805, p < 0.001 ^§^; *post hoc*: MDD+CM *vs* HC, p < 0.001; MDD *vs* HC, p = 0.002; MDD+CM *vs* MDD, p = 0.566
DERS–awareness	16 (13.5-19) *	13 (10-18)	12 (9-15.5)	F= 4.423; p = 0.016 ^†^; *post hoc*: MDD+CM *vs* HC, p = 0.018; MDD *vs* HC, p = 1.000; MDD+CM *vs* MDD, p = 0.122
DERS–strategies	28 (22.5-34.5) *** ^#^	23 (17.5-28.25) ***	13 (9.5-17)	F= 34.283; p < 0.001 ^†^; *post hoc*: MDD+CM *vs* HC, p < 0.001; MDD *vs* HC, p < 0.001; MDD+CM *vs* MDD, p = 0.014
DERS–clarity	15 (10-19) ***	9 (6.75-12)	8 (6-9.5)	χ^2^ = 15.478, p < 0.001 ^§^; *post hoc*: MDD+CM *vs* HC, p < 0.001; MDD *vs* HC, p = 0.442; MDD+CM *vs* MDD, p = 0.064
TAS sum	61 (47-68.5) ***	49.5 (44-55.25) **	38 (31-42)	F= 15.124; p < 0.001 ^&^; *post hoc*: MDD+CM *vs* HC, p < 0.001; MDD *vs* HC, p = 0.003; MDD+CM *vs* MDD, p = 0.141
TAS–difficulty identifying feelings	22 (17.5-25) ***	17 (11.75-19.25) *	12 (8-15.50)	χ^2^ = 25.104, p < 0.001 ^§^; *post hoc*: MDD+CM *vs* HC, p < 0.001; MDD *vs* HC, p = 0.035; MDD+CM *vs* MDD, p = 0.067
TAS–difficulty describing feelings	13 (11-19) **	13.5 (10.75-17)	10 (6.5-12)	F= 7.129; p = 0.002 ^†^; *post hoc*: MDD+CM *vs* HC, p = 0.001; MDD *vs* HC, p = 0.054; MDD+CM *vs* MDD, p = 0.802
TAS–external oriented thinking	20 (16.5-25) *	19 (18-21.25) *	17 (13-20)	F= 6.391; p < 0.001 ^&^; *post hoc*: MDD+CM *vs* HC, p = 0.011; MDD *vs* HC, p = 0.016; MDD+CM *vs* MDD, p = 0.737
RMET sum	24 (22-27.5)	27 (24.25-28.25)	27 (24-29)	F= 2.579; p = 0.085 ^†^
RMET emotional sum	13 (11-15.5)	15 (14-16)	14 (12-16)	F= 2.670; p = 0.078 ^†^
RMET neutral faces	11 (10.5-14)	11.5 (11-14)	12 (11-14.5)	F= 1.221; p = 0.303 ^†^
RMET negative emotion	7 (5-8)	8 (7-9)	8 (7-9)	χ^2^ = 5.342, p = 0.069 ^§^
RMET positive emotion	6 (5-7.5)	7 (6-7.25)	7 (5-8)	χ^2^ = 1.109, p = 0.574 ^§^

Data are expressed as median (interquartile range).

Benjamini-Hochberg correction was not applied here, because this approach can increase the type II error and may result in elimination of the group-specific differences.

MDD+CM, major depressive disorder with childhood maltreatment; MDD, major depressive disorder; HC, healthy control; CERQ, Cognitive Emotion Regulation Scale; CERQ maladaptive sum, sum of the Self-blame, Rumination, Catastrophizing, and Blaming others scores; CERQ adaptive, sum of the Acceptance, Positive refocusing, Refocus on planning, Positive Reappraisal and Putting into perspective scores; DERS, Difficulties in Emotion Regulation Scale; TAS, Toronto Alexithymia Scale; RMET, Reading Mind in the Eyes Test; *vs*, versus.

^†^One-way ANOVA with Bonferroni *post hoc* test; ^&^One-way ANOVA with Dunnett’s T3 *post hoc* test ^§^Kruskal-Wallis H test with Dunn’s pairwise *post hoc* test; *p < 0.05, **p < 0.01, ***p < 0.001 *versus* the healthy controls; ^#^p < 0.05 *versus* the MDD group.

Assessment with the Cognitive Emotion Regulation Questionnaire revealed that compared to the other two groups maltreated individuals reached the highest scores in the CERQ maladaptive sum and the lowest scores in the CERQ adaptive sum (**Table 3**). Maltreated patients had significantly different scores compared to controls in almost all subscales. Similarly, non-maltreated depressed individuals had significantly different scores compared to controls in most of the subscales and they also reached significantly higher scores in the CERQ maladaptive sum and significantly lower scores in the CERQ adaptive sum compared to controls (**Table 3**).

Results of the Difficulties in Emotion Regulation Scale indicated that maltreated individuals had the most severe difficulties with emotion regulation. They had the highest scores in DERS sum, and in all subscales, and they were always significantly different compared to controls (**Table 3**). Non-maltreated depressed patients also had difficulties in emotion regulation as they reached significantly higher scores in DERS sum and in most of the subscales compared to controls (**Table 3**).

Results of the Toronto Alexithymia Scale indicated that maltreated individuals reached the highest scores, and they were always significantly different compared to controls (**Table 3**). Eleven maltreated depressed individuals had equal to or greater than 61 TAS sum scores indicating alexithymia. Non-maltreated depressed patients had significantly higher TAS sum scores compared to controls and in one TAS subscale (Difficulty Identifying Feelings), they were also different compared to controls. Among the non-maltreated depressed patients, there were two subjects who had 61≤ TAS sum scores indicating alexithymia.

The Reading the Mind in the Eyes Test revealed no difference between the three experimental groups (**Table 3**).

### 3.4 Positive correlations between hippocampal subfield volumes and results of the psychological tests

The positive correlations between brain area volumes and results of the psychological tests are presented in **Table 4**. We report here only the statistically significant findings.

**Table 4:** Between-group interaction effects and positive correlations between the psychological parameters and hippocampal subfield and amygdala volumes.

Group	Clinical variable	Region	Interaction p-value*	Correlation p-value*	ß value
MDD+CM	CERQ - rumination	right CA3	**0.024**	0.035	0.365
	CERQ - positive reappraisal	left presubiculum	**0.005**	0.002	0.544
	CTQ - emotional abuse	right amygdala	0.022	0.006	0.440
		left amygdala	0.032	0.004	0.464
MDD	RMET - positive emotion	left presubiculum	0.045	0.019	0.432
	CTQ - emotional neglect	right CA3	0.053	0.040	0.480
HC	RMET - neutral faces	right CA1	**0.019**	0.010	0.523
		right subiculum	**0.001**	<0.001	0.660
		left molekular layer	**0.037**	0.026	0.432
		left subiculum	**0.017**	0.004	0.544

Bold p-values indicate when the interactions remained significant after controlling for multiple comparisons using the Benjamini-Hochberg method with q=0.15.

MDD+CM, major depressive disorder with childhood maltreatment; MDD, major depressive disorder; HC, healthy control; CERQ, Cognitive Emotion Regulation Questionnaire; CTQ, Childhood Trauma Questionnaire; RMET, Reading the Mind in the Eyes test; CA, Cornu Ammonis.

*Corrected for age and estimated total intracranial volume.

In maltreated depressed patients, the scores reached in the rumination subscale of the CERQ test showed positive correlation with the volume of the right CA3 area. Notably, the right CA3 area had no volume shrinkage in these patients (**Table 2**). Furthermore, in maltreated patients, the low scores of the positive reappraisal subscale of the CERQ test were associated with the volume reduction of the left presubiculum (**Table 4**).

In non-maltreated depressed patients (MDD group), results of recognizing positive emotions in the RMET test showed positive correlation with the volume of the left presubiculum. Furthermore, results of the emotional neglect subscale of the CTQ test showed positive correlation with the volume of the right CA3 area. However, none of these two results remain significant after controlling for multiple comparisons using the Benjamini-Hochberg method with q=0.15 (**Table 4**).

In the control group, the results of recognizing neutral faces in the RMET test showed positive correlations with several hippocampal subfield volumes, namely with the volumes of the right CA1, right subiculum, left molecular layer, and left subiculum (**Table 4**).

### 3.5 Negative correlations between the hippocampal subfield volumes and results of the psychological tests

The negative correlations between brain area volumes and results of the psychological tests are presented in **Table 5**. We report here only the statistically significant findings.

**Table 5:** Between-group interaction effects and negative correlations between the psychological parameters and hippocampal subfield and amygdala volumes.

Group	Clinical variable	Region	Interaction p-value*	Correlation p-value*	ß value
MDD	CERQ - rumination	right hippocampus	**0.016**	0.004	-0.510
		right GCL-ML-DG	**0.023**	0.011	-0.456
		right molecular layer	**0.033**	0.007	-0.528
		right CA4	**0.035**	0.018	-0.435
		right CA1	**0.020**	0.004	-0.530
		right amygdala	**0.019**	0.001	-0.599
HC	DERS - awareness	right hippocampal tail	**0.048**	0.030	-0.574
		left subiculum	**0.028**	0.017	-0.547
	CERQ - acceptance	right CA3	0.022	0.009	-0.589

Bold p-values indicate interactions that remained significant after controlling for multiple comparisons using the Benjamini-Hochberg method with q=0.15.

MDD, major depressive disorder; HC, healthy control; CERQ, Cognitive Emotion Regulation Questionnaire; DERS, Difficulties in Emotion Regulation Scale; GCL-ML-DG, granule cell layer and molecular layer of the dentate gyrus; CA, Cornu Ammonis.

*Corrected for age and estimated total intracranial volume.

In maltreated depressed patients, we did not find any negative correlations between the results of the psychological tests and brain area volumes.

In the non-maltreated MDD patients, the scores reached in the rumination subscale of the CERQ test showed negative correlations with the volumes of several subfields of the right hippocampus i.e. the GCL-ML-DG, molecular layer, CA4, CA1, and the entire right hippocampus (**Table 5**).

In the control group, results of the awareness subscale of the DERS test showed negative correlation with the volumes of the right hippocampal tail and left subiculum. Furthermore, in control subjects results of the acceptance subscale of the CERQ test showed negative correlation with the volume of the right CA3, but this result did not remain significant after controlling for multiple comparisons using the Benjamini-Hochberg method with q=0.15 (**Table 5**).

### 3.6 Correlations between amygdala volumes and the results of the psychological tests

In case of the amygdala, we found very few significant correlations. In maltreated individuals, the scores of the emotional abuse subscale in the CTQ test were positively associated with volume of the bilateral amygdala. However, this result did not remain significant after controlling for multiple comparisons using the Benjamini-Hochberg method with q=0.15 (**Table 4**). Furthermore, in non-maltreated depressed patients, the scores of the rumination subscale in the CERQ were associated with greater volume shrinkage of the right amygdala (**Table 5**).

## 4 Discussion

The present study was designed to investigate the associations between emotion processing and volumes of hippocampal subfields and amygdala. We focused on depressed patients since emotional dysregulation and hippocampal volume shrinkage are key characteristics of this population. Our first hypothesis was that depressed patients, particularly those who experienced childhood maltreatment, would demonstrate reduced hippocampal volumes. We were only able to partially confirm this. While we observed a clear trend of volume decrease in the depressed patients, this difference did not achieve statistical significance. It is noteworthy that volume shrinkage was more prominent in those with a history of maltreatment and affected both the hippocampus and the amygdala. We emphasize these non-significant volumetric differences between the groups because the absence of volumetric variation would undermine the meaningful interpretation of the volumetric correlations. Furthermore, our findings revealed that maltreated depressed subjects showed a 6% reduction in hippocampal volume across both hemispheres. In comparison, meta-analytic studies focusing on depressed patients have reported a reduction of 8% in left hippocampal volume and 10% in right hippocampal volume (87), which is to some extent comparable to our data. It is important to note, however, that the relatively low sample size of our study may have contributed to our inability to detect statistically significant differences between the groups.

Our second hypothesis was that depressed patients will exhibit maladaptive behaviors when assessed with the psychological tests and this hypothesis was clearly confirmed. Maltreated depressed patients had the most pronounced difficulties in emotion processing. Emotional dysregulation was characteristic of them as they were significantly different compared to controls in all subscales of the DERS test. Maltreated individuals also presented numerous difficulties in cognitive coping as they were significantly different compared to controls in almost all subscales of the CERQ test. Eleven maltreated depressed individuals had alexithymia, and the maltreated group was significantly different compared to controls in all subscales of the TAS test. Non-maltreated depressed patients also had severe problems with emotional regulation, coping behavior and alexithymia, because they had significantly different scores compared to controls in almost all subscales of the DERS, CERQ and TAS tests.

However, we could not confirm our third hypothesis, namely that the maladaptive behaviors will correlate with the hippocampal and/or amygdala volume shrinkage. We expected numerous negative correlations between the high scores of the psychological measures and the volume shrinkage of the hippocampal subfields, but we found only a few. In maltreated depressed patients, we did not find any negative correlations, and in the non-maltreated depressed patients, only the results of the rumination subscale of the CERQ test showed negative correlations with volumes of the right hippocampal subfields. Surprisingly, not even the results of the CTQ test had any negative correlations with brain area volumes. As a matter of fact, in maltreated individuals, the high scores of the emotional abuse subscale of the CTQ test were associated with volume of the bilateral amygdala. Furthermore, while maltreated patients had significantly higher scores in the TAS and 11 maltreated individuals had alexithymia, these abnormalities did not correlate with any brain area volumes investigated by us.

### 4.1 Correlation between MRI data and emotional processing

Numerous studies have used *in vivo* magnetic resonance imaging to examine hippocampal volume changes in subjects suffering from major depressive disorder, or in relation to adverse childhood experiences (17, 25, 30, 87–91). While the meta-analytic studies reveal a significant hippocampal volume loss of 8-10%, the results of the individual studies have been inconsistent and negative findings are not without precedent (87, 88, 92). The exact cellular changes responsible for the hippocampal volume loss are not fully understood, neuronal loss, dendritic reorganization, reduced adult neurogenesis and glial changes have all been implicated (93). A widely held view is that the stress-induced activation of the HPA-axis results in elevated glucocorticoid levels, which then initiates a cascade of neurotoxic – or at least neuroplastic – events in the brain, resulting in gross volume decrease (94–96). Based on this line of thinking, one may conclude that the hippocampal volume shrinkage is linked to the maladaptive emotional and/or cognitive behavior typical for depressed patients. Indeed, there is evidence for such a consequence, for example a study reported that in depressed patients the hippocampal volume loss correlated with executive dysfunctions (97). A more recent longitudinal study of depressed youth could also link hippocampal volume with emotion regulation and episodic memory impairment (34). Results of our present study could not however, substantiate these earlier findings.

The notion that volumetric changes in limbic structures can be linked to functional impairments has a long tradition (e.g. 15, 97, 98). Nevertheless, studies that have directly examined the structure-behavior relationship have so far yielded ambiguous results (18, 99, 100). The factors contributing to changes in brain area volume may be more complex or variable, rendering the correlation between brain structure and behavior challenging. Therefore, correlating functional neuroimaging data with complex psychological functioning may yield more consistent results.

Several factors may explain our inability to demonstrate an association between maladaptive emotional behavior and hippocampal subfield or amygdala volumes. One significant consideration is neuroplasticity, a key characteristic of the human brain. Volumetric changes may occur more rapidly and with greater variability than previously assumed (101). Furthermore, individual variability in brain development (102), hippocampal volume (21, 103), and emotional brain network topology (104) may provide another explanation. Additionally, functional reorganization (105) and structural resilience (106) following traumatic experiences may further elucidate the absence of significant results in our study.

### 4.2 Associations between difficulties in emotion regulation and volumes of hippocampal subfields and amygdala

Subjects of the present study were assessed with five psychological tests and the rumination subscale of the CERQ test was the one which had the largest number of correlations with hippocampal subfield volumes. This was reassuring since the hippocampus has been implicated in the regulation of stress-coping strategies (107, 108). The CERQ test has been constructed to identify the cognitive coping strategies in response to stressful life events (35). A recent study found a few positive correlations with some subscales of the CERQ test (e.g. catastrophizing, rumination, refocus on planning and positive refocusing) in healthy individuals, but none these correlations remained significant after correction for multiple analyses (19).

We also found a few negative correlations between results of the awareness subscale of the DERS test and hippocampal subfield volumes of control subjects. Only a few studies investigated the relationship between hippocampal, or amygdala volumes and emotion regulation difficulties. One study reported a strong relationship between emotion regulation and hippocampal volume (34), while another found that prolonged orphanage rearing was associated with atypically large amygdala volume and difficulties in emotion regulation (109).

In case of the RMET test, we found no difference between the groups, but within-group correlation analysis revealed that in the control group there were numerous positive correlations between hippocampal subfield volumes and scores of the RMET-neutral-faces subscale. Numerous MRI studies have been performed to relate performance in the RMET test with results of structural brain imaging and there is evidence that larger amygdala/hippocampal volumes are associated with better performance in the RMET test (52, 54).

In case of the TAS test, we could not find any correlations with brain area volumes. In the literature, there are numerous imaging studies which investigated the association between alexithymia and gray matter volumes, but most studies yielded inconsistent findings (40). Results of a recent meta-analysis indicates that the volumes of the left insula, left amygdala, orbital frontal cortex and striatum is consistently smaller in people with high levels of alexithymia (40). However, our present data could not replicate these findings.

Our present study has yielded a few counterintuitive correlations. Notably, we observed that individuals who reported higher instances of emotional abuse exhibited larger volumes of their bilateral amygdalae. Although this result did not remain significant after controlling for multiple comparisons, it stands in contrast to previous research, which typically associates childhood emotional abuse with reduced amygdala volumes (110–112). As discussed in chapter 4.1, potential explanations for these contradictory results may include individual differences in brain development, sample variability, or the presence of statistical noise.

### 4.3 Limitations

As with the majority of studies, the current research is subject to several limitations. A major limitation is the relatively low sample size. To circumvent this issue, participants were meticulously selected to ensure matching groups in terms of age, gender, depression severity, and IQ. The relatively small sample size is likely a contributing factor to our inability to detect statistically significant volumetric differences among the three examined groups. A formal power analysis calculated by G*Power (version 3.1.9.4) indicated that a minimum total sample size of 153 participants (51 per group) would be required to detect statistically significant volumetric differences across the three groups in hippocampal subfields and the amygdala, based on assumed effect size of 0.322 and a statistical power of 0.9508. Further limitations are the cross-sectional design of the study and the retrospective assessment of childhood maltreatment, which was conducted with a self-report questionnaire that lacks complete objectivity. The cross-sectional nature of our study coupled with the retrospective self-reporting of childhood abuse, limits our ability to establish causal relationships between childhood maltreatment, brain morphology, and emotional regulation. Longitudinal studies are necessary to clarify such causal relationships, and indeed, there have been efforts to verify such causalities. A recent longitudinal study demonstrated that childhood maltreatment is associated with a persistent reduction of hippocampal volume in children and adolescents (113). Similarly, longitudinal studies could prove that experiencing childhood maltreatment is related to emotion dysregulation (114, 115).

The segmentation of the hippocampal subfields and the amygdala was based on only T1-weighted images, as employed in previous studies (116–118). For this reason, finding related to the volumes of GCL-ML-DG, CA4 and molecular layer must be interpreted with caution. Additionally, a limitation is that the amygdala was not segmented into nuclei. This limitation arose because we began the data analysis with the latest available version of the Freesurfer software (version 6.0), which does not include the segmentation of amygdala subregions. As a result, segmentation was confined to the entire amygdala without any division into further subregions. Future studies that employ high-resolution T2-weighted images and more recent versions of FreeSurfer software (beyond version 6.0) are warranted to achieve more detailed segmentation of the amygdala. Finally, this study relied only on structural neuroimaging data, whereas correlating functional neuroimaging findings with strategies of emotion regulation in maltreated and non-maltreated patients with major depression may yield more robust and informative results.

### 4.4 Conclusion

We report here that depressed patients with or without childhood maltreatment exhibit a modest reduction in hippocampal volume. Moreover, these individuals also display pronounced difficulties in emotion regulation. We demonstrate here a few associations between hippocampal subfield and amygdala volumes and disturbances in emotional processing. However, we could not detect the expected negative correlations between maladaptive behavior and hippocampal/amygdala volume shrinkage. Our present data suggest that correlating volumes of specific brain regions with complex psychological functions may not yield convincing results. Consequently, future research should prioritize functional neuroimaging methods, such as assessments of neural activity or functional connectivity, over structural data, like volumetric measurements, when investigating complex emotional phenomena.

## Data Availability

The raw data supporting the conclusions of this article will be made available by the authors, without undue reservation.
